# Can Enterococcus faecium prevent NEC in preterm infants?: A systematic review and meta-analysis

**DOI:** 10.1097/MD.0000000000034787

**Published:** 2023-08-11

**Authors:** Guangguo Men, Lili Wang, Xudan Lu, Gang Wen, Qin Lü

**Affiliations:** a Neonatal Intensive Care Unit, Ningbo Women and Children’s Hospital, Ningbo, China; b Department of Neonatology, Dong’e Hospital of Traditional Chinese Medicine, Liaocheng, Shandong, China; c Department of Neonatology, Ningbo Women and Children’s Hospital, Ningbo, China; d Department of Pediatric Surgery, Ningbo Women and Children’s Hospital, Ningbo, China.

**Keywords:** Enterococcus faecium, necrotizing enterocolitis, preterm infants

## Abstract

Previous some systematic reviews reported that probiotics may benefit the prevention of NEC in preterm infants. But dissimilar bacterial strains and taxa used in included studies possibly result in bias. There is not a rounded systematic review which has estimated the benefit and safety of Enterococcus faecium to prevent NEC in preterm infants to date before we conducted.

**Methods:**

This systematic review of randomized controlled trials and retrospective studies analyzing the benefit of Enterococcus faecium to prevent NEC in preterm infants was performed using PubMed, Web of Science, Cochrane Library, EMBASE, Wanfang data and China National Knowledge Infrastructure databases from inception to April 14, 2023. The search terms were “preterm” AND “necrotizing enterocolitis” AND “Enterococcus faecium OR probiotics.” Studies reporting NEC involving preterm infants who were given Enterococcus faecium were included in this systematic review. A sensitivity analysis was conducted to assess the stability of results. A funnel plot was generated to identify publication bias. Two authors appraised studies quality and extracted data independently. This work has been reported according with preferred reporting items for systematic reviews and meta-analyses and assessing the methodological quality of systematic reviews. Statistical analysis was conducted using Review Manager 5.3 software. Risk ratio (RR) with 95% confidence intervals (CI) was calculated and analyzed.

**Results:**

Seven studies (N = 1487 participants) were included in this systematic review, and 6 randomized, controlled trials (N = 1237 participants) were included in the meta-analysis. Comparing with the control groups, the Enterococcus faecium groups had a significant decline in the incidence of NEC Bell stage II or higher (RR: 0.3138, 95% CI: 0.1983–0.4965; *P* < .00001; 6 studies, n = 1237) and infection (RR: 0.4818, 95% CI: 0.2950–0.7869; *P* = .004; 3 studies, n = 710).

**Conclusions:**

Enterococcus faecium is effective and safe in preventing NEC (Bell stage II or higher) in preterm infants. But all studies included came from China. The dosages and durations of taking Enterococcus faecium were various.

## 1. Introduction

Necrotizing enterocolitis (NEC) is a significant issue for very preterm infants with gestational age (GA) < 32 weeks, especially those with GA < 28 weeks or low birth weight (BW).^[1]^ The disease accounts for 1% to 5% of inpatient admissions to neonatal intensive care units (NICU), with a mortality rate of 15% to 30%. NEC occurs in about 7% to 10% of preterm infants with very low birth weight (BW < 1500 g) or GA < 32 weeks.^[2–4]^ NEC is characterized by signs of abdominal distention, bloody stools, bilious emesis, apnea, lethargy, bradycardia and the specific finding of discoloration and abdominal tenderness.^[5]^ The health burden associated with ≥ Stage II NEC in preterm infants is significant.^[6]^ The role of the microbiome in NEC has been explored extensively in the last 20 years.^[6,7]^ Probiotics are beneficial for preterm infants to prevent NEC.^[8–13]^

The pathogeny and pathogenesis of NEC is inconclusive but should be related to barrier function, immature intestinal motility, abnormal bacterial colonization, digestion, and innate immunity. So far, the cause is still unclear, despite many molecular biology and microbiology studies of premature infants with NEC.^[14]^ Claud and Walker (2001)^[15]^ thought that the occurrence of NEC should be associated with improper microbial colonization. Sanders shared mechanisms among probiotic taxa to explain the probiotic claims. They provide some scientific evidence on shared mechanisms of general probiotic strains that are genus-specific, species-specific or sub-species-specific.^[16]^

The severity of NEC is rated by the Bell staging criteria. Stage I is the “suspect” stage, characterized by gastrointestinal manifestations and abdominal distension on radiograph. Stage II, the “definite” stage, is marked by gastrointestinal bleeding and multiple radiographic findings including significant intestinal distention, ileus, edema, pneumatosis intestinalis, and portal vein gas. Finally, Stage III is the “advanced” stage of the disease, progressing to rapid deterioration, bowel necrosis, septic shock, gastrointestinal hemorrhage, and pneumoperitoneum on radiograph. Stage III requires operative intervention for resection of necrotic intestine. The Bell staging criteria is widely used to assess the state of NEC.^[17]^ Although the criteria have been periodically modified and despite criticism that it is outdated, the use of Bell staging criteria remains standard practice.^[18,19]^ Many research showed that probiotics were beneficial for preterm infants to prevention and treatment of NEC Bell stage II or higher. Athalye-Jape and Rao (2017) indicated that probiotics significantly declined the morbidity rate of NEC via improvement in inflammation and immunity, intestinal barrier function, tissue injury, and gut microflora dysbiosis.^[20]^ Some clinical tests have achieved the assessment of probiotics to prevent NEC in preterm infants. However, the type of probiotic, the dosage and the duration of the intervention used to prevent NEC in preterm infants were different in the included studies. The probiotic strains used to prevent and treat NEC in the world are Enterococcus faecium, Bacillus subtilis, Bifidobacterium, Lactobacillus, Streptococcus, and so on. However, in China, Enterococcus faecium, and Bifidobacterium are commonly used in clinical practice. The adverse events of Enterococcus faecium, including sepsis and death were not reported. In addition, the incidence of NEC has not been summarized and evaluated.

Some systematic reviews and meta-analyses reported that probiotics may benefit the prevention of NEC in preterm infants.^[1,17]^ However, the different strains used in the studies may lead to bias. Up to now, no systematic review has evaluated the benefits and safety of Enterococcus faecium in preventing NEC in preterm infants. So, we conducted this systematic review including 6 randomized and 1 non-randomized controlled trials, with 1487 participants to study the benefit of Enterococcus faecium on the morbidity of NEC in preterm infants. In addition, adverse events including sepsis and death would be analyzed to assess the safety of Enterococcus faecium in preterm infants.

## 2. Methods

### 2.1. Inclusion criteria and exclusion criteria

#### 2.1.1. Inclusion criteria.

We defined the inclusion of eligible studies on the basis of the patients, intervention, control, outcome, study criteria. The inclusion criteria were as follows: Participants were newborns with GA < 37 weeks; Administration of Enterococcus faecium for preventing NEC; Study was designed as randomized controlled trial (RCT) or controlled clinical trials (CCT); Outcomes including NEC stage II or higher (Bell staging criteria),^[21]^ sepsis or infection, and mortality.

#### 2.1.2. Exclusion criteria.

In order to ensure the accuracy of this systematic review, the followings were excluded: Animal tests; Reviews, abstracts, expert opinions, case reports, letters, and editorials; Studies without sufficient data; Studies including infants who had other congenital disease.

### 2.2. Outcome

#### 2.2.1. Primary outcome.

The primary outcome is the incidence of NEC.

#### 2.2.2. Secondary outcomes.

The secondary outcomes included the incidence of sepsis, infection and mortality.

### 2.3. Search strategy

A search was conducted in PubMed, Web of Science, Cochrane Library, EMBASE, Wanfang data and China National Knowledge Infrastructure databases for studies published before April 14, 2023. We used PubMed medical subject heading terms with Boolean operators as comprehensively as possible: (infant*[ti] OR baby[ti] OR babies[ti] OR preterm[ti] OR preemie*[ti] OR newborn*[ti] OR neonate*[ti]) AND (“necrotizing enterocolitis” OR “necrotizing enterocolitis” OR “sepsis” OR “death”) AND (“Enterococcus faecium” OR “probiotics”). Other relevant terms, including references of some articles, were also searched. There are no restrictions on the language of retrieval. Search process was done on April 14, 2023.

Two reviewers independently retrieved the literature. After excluding duplicate literature, the reviewers screened the studies by title and abstract, following the inclusion criteria, participant type, intervention methods, and outcome reported. All conflicts were resolved with the intervention of the third or more authors. All full texts of related articles were read carefully and thoroughly for accurate assessment.

### 2.4. Risk of bias assessment and quality evaluation

Two reviewers independently evaluated the quality of every study included. Risk of bias was estimated with the Jadad score^[22]^ with scores between 0 (very high risk of bias) and 5 (very low risk). All the scores were presented in Table 1. High-quality research was defined as those with a Jadad score ≥ 3. Furthermore, the risk of bias of every study and the risk of bias across all studies were evaluated and visualized with figures generated by RevMan 5.3 software.^[30]^

**Table 1:** The characteristics of included studies for systematic review of efficacy and safety of Enterococcus faecium.

Study/yr	Country	Design	BW/GA	Probiotic interventions	Dosage and duration	E/C (No. of NEC stage ≥ II)	E/C (total No.)	Outcomes	Jadad score
Deng ZQ 2018^[23]^	China	RCT	Group experiment: GA: (31.97 ± 2.42) wk; BW: (1688 ± 336) g; Group control: GA: (31.95 ± 2.55) wk; BW: (1679 ± 353) g	Medilac-Vita	BW: <1500 g, 0.33 g/dose; 1500–2000 g,0.5 g/dose; >2000 g, 1 g/dose; bid. 2 wk	2/8	67/67	NEC, infection	3
Fu HT 2012^[24]^	China	CCT	All infants as follow: GA: <32 wk, n = 12; 32~35 wk, n = 6; >35 wk, n = 4; BW: <1500 g, n = 15; 1500~2000 g, n = 6; >2000 g, n = 1	Medilac-Vita	0.5 g/dose, bid	4/18	126/124	NEC	0
Gan JL 2013^[25]^	China	RCT	Group experiment: BW: <1500 g, n = 166; ≥1500g, n = 120; GA: 28~31^+6^ wk, n = 57;32~34^+6^ wk, n = 229; Group control: BW: <1500 g, n = 112; ≥1500 g, n = 98; GA: 28~31^+6^ wk, n = 36;32~34^+6^ wk, n = 174	Medilac-Vita	0.5 g/dose, bid	15/22	286/210	NEC, infection, feeding intolerance	2
Lin WQ 2018^[26]^	China	RCT	Group experiment: BW: 1.86 ± 0.48 kg; GA 33.01 ± 2.48 wk; Group control: BW: 1.84 ± 0.41 kg; GA: 32.21 ± 3.21 wk	Medilac-Vita	0.33 g/dose, q12 h, 14 d	1/6	46/46	NEC, hyperbilirubinemia of newborn	1
Lu L 2008^[27]^	China	RCT	Group experiment: BW: 1530 ± 352 g, GA: 29 ± 4 wk; Group control: BW 1580 ± 343 g; GA 30 ± 3 wk	Medilac-Vita	0.33 g/dose, tid, 7 d	0/1	40/40	NEC, mortality, IVH	2
Zhai M 2010^[28]^	China	RCT	N/A. but GA of infants with NEC as follow: Group experiment: <32 wk, n = 2; 32–37 wk, n = 2; Group control: <32 wk, n = 8; 32–37 wk, n = 11	Medilac-Vita	1 g/d	5/19	182/173	NEC	2
Zhang Q 2019^[29]^	China	RCT	Group experiment: GA (30.6 ± 1.6) wk; BW: (1.5 ± 0.2) kg; Group control: GA: (30.5 ± 1.4) wk; BW: (1.6 ± 0.1) kg	Medilac-Vita	0.5 g/dose, bid, 2 wk	1/10	40/40	NEC, infection	3

Medilac-Vita, combined bacillus subtilis and enterococcus faecium granules with multi-vitamine, Live = It contains 135 million Enterococcus faecium and 15 million Bacillus subtilis every gram.

BW = birth weight, CCT = controlled clinical trials, E/C = group experiment/group control, GA = gestational age, IVH = intraventricular hemorrhage, N/A = not available, NEC = necrotizing enterocolitis, RCT = randomized controlled trial.

### 2.5. Data extraction

Once the screening process was completed, the 2 reviewers independently extracted the data of selected articles and recorded in a prepared form. Any conflict was resolved by discussing. The extracted contents included author, year of publication, demographic characteristics, study design, number of participants, GA, BW, intervention method, numbers of NEC (Bell stage II or higher, Bell staging criteria),^[21,31]^ outcomes, and Jadad score. The data were analyzed by the statistical software RevMan 5.3.^[30]^ We would contact the author as much as possible by email if the data was not accessible, but the study would be excluded from the meta-analysis if the author couldn’t be contacted or the data could not be acquired.

### 2.6. Statistical analysis

RevMan 5.3 software^[30]^ was used for statistical analysis in this meta-analysis. The pooled risk ratio (RR) was evaluated data (incidence of NEC, sepsis and mortality) by a random-effects model (significant heterogeneity, *P* < .05) or a fixed-effects model (the heterogeneity was not significant, *P* > .05). The effect sizes were shown as RR with 95% confidence intervals (CI), and a forest plot was used to display results. The results of heterogeneity of the studies were evaluated by the chi-squared based Q test and I^2^.^[32]^ *P* < .05 or I^2^ > 50% was deemed statistically significant heterogeneity.^[33,34]^ General descriptive analysis was only performed if the source of heterogeneity was not found. Funnel plots were used to represent publication bias. A sensitivity analysis was conducted to assess the stability of the combined results. We conducted a subgroup analysis of the pooled result of NEC according to Jadad score (1–2, 3). For outcomes that could not be meta-analyzed, but were more important, such as mortality, we adopted narrative reporting.

The work has been reviewed in accordance with the updated preferred reporting items for systematic reviews and meta-analyses 2020 guideline^[35]^ and the assessing the methodological quality of systematic reviews 2 guideline.^[36]^

## 3. Results

### 3.1. Characteristics of studies

**Figure 1.**
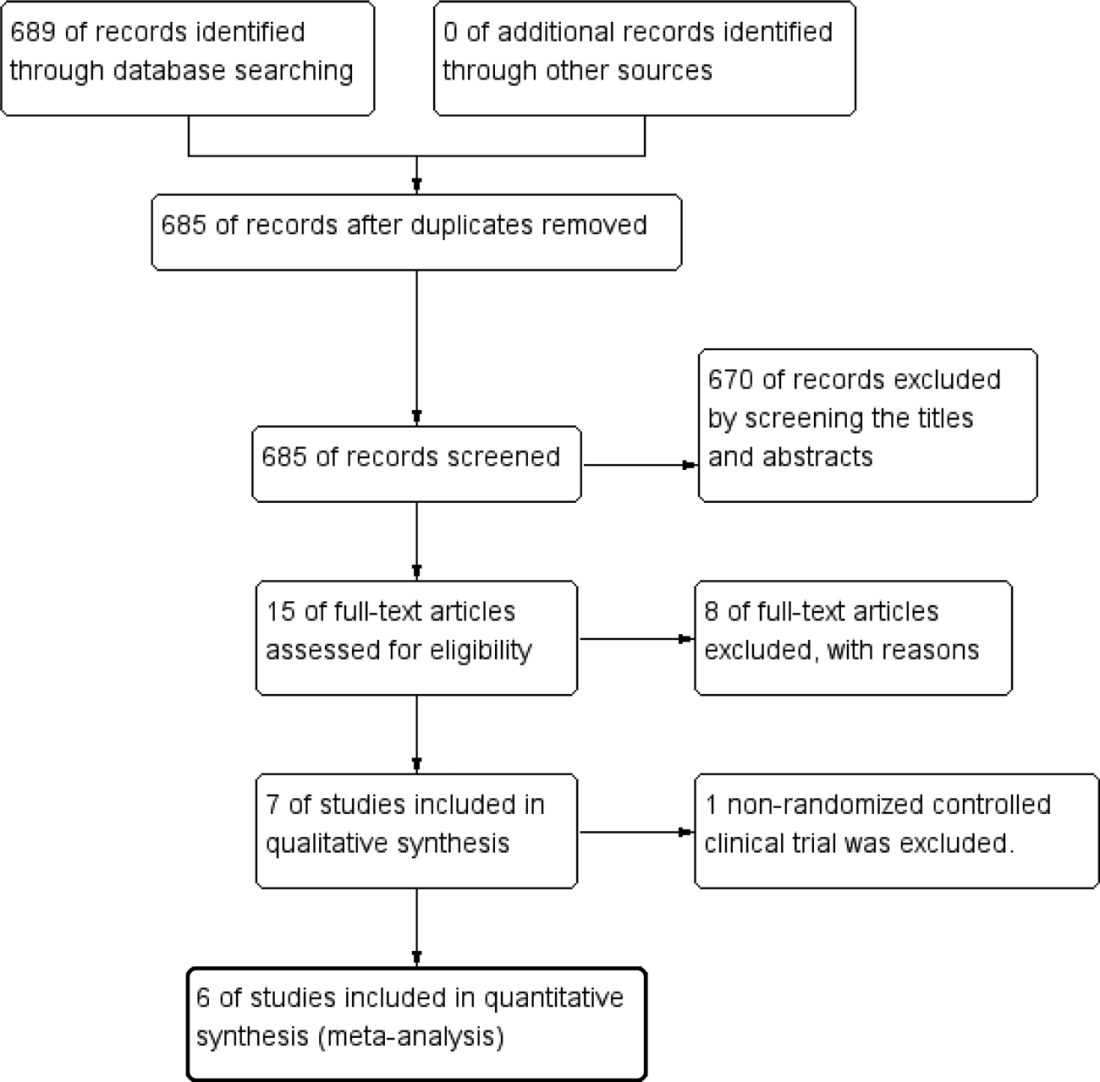
Literature search and screening process flow chart. Flow diagram following the preferred reporting items for systematic reviews and meta-analyses (PRISMA) template of the search strategy for efficacy and safety of Enterococcus faecium in preventing necrotizing enterocolitis (NEC) in preterm infants.

After retrieving all the titles and abstracts of 685 originally searched articles, 670 were removed and 15 were selected for reviewing full texts. After reviewing full texts of the 15 articles, 8 were excluded, and eventually 7 studies (N = 1487 participants)^[23–29]^ were included in this systematic review. Of these included studies, 6 are RCTs^[23,25–29]^ and 1 is CCT.^[24]^ In order to ensure the accuracy of the study, 6 RCTs (N = 1237 participants) were finally included in this meta-analysis.^[23,25–29]^ The sample sizes ranged from 80 to 496. All 7 studies used Enterococcus faecium to prevent NEC in preterm infants. The characteristics of including studies are shown in Table 1. We present the following studies in accordance with the preferred reporting items for systematic reviews and meta-analyses reporting checklist. The particular of searching process and the summary of these studies are listed in flow diagram (Fig. 1).

### 3.2. Quality assessment

**Figure 2.**
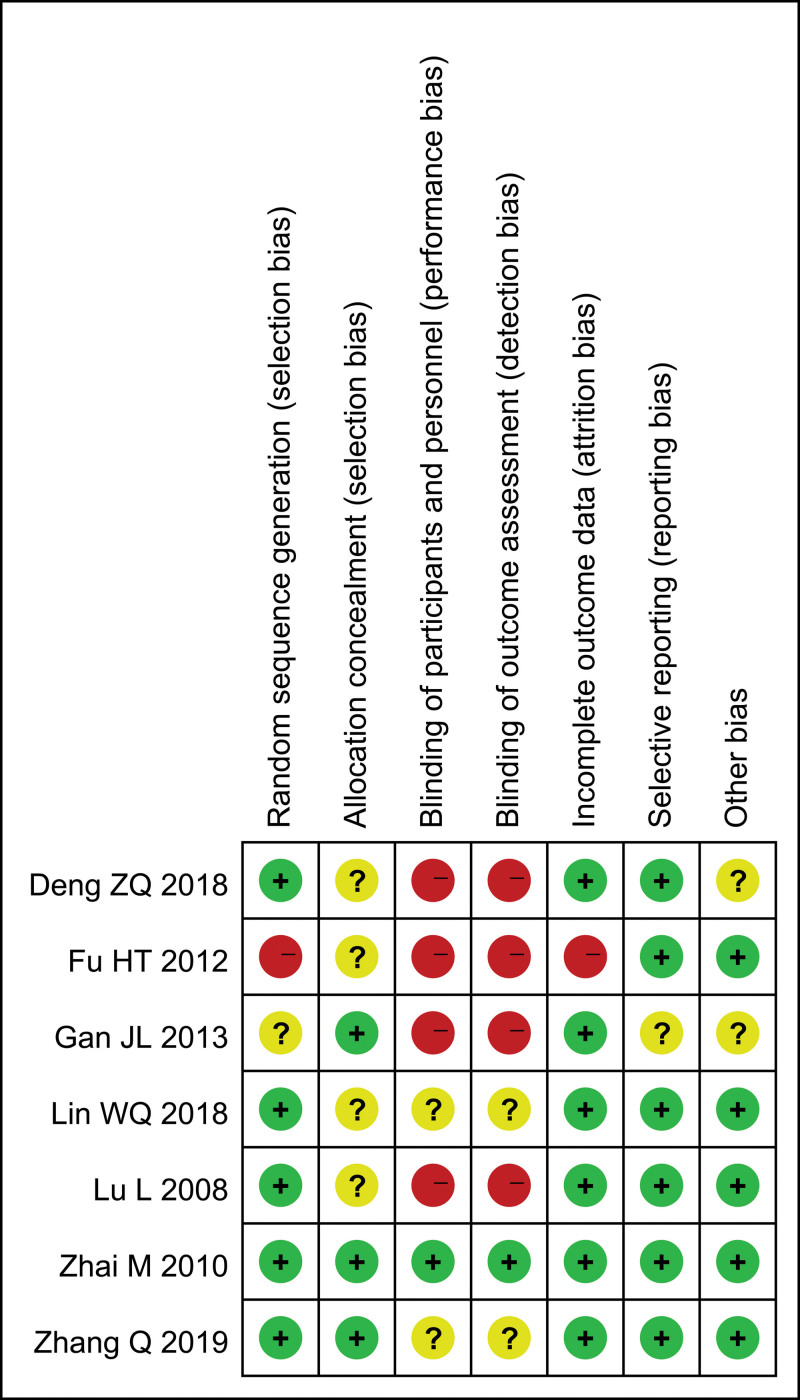
Risk of bias summary: review authors’ judgements about each risk of bias item for each included study.

**Figure 3.**
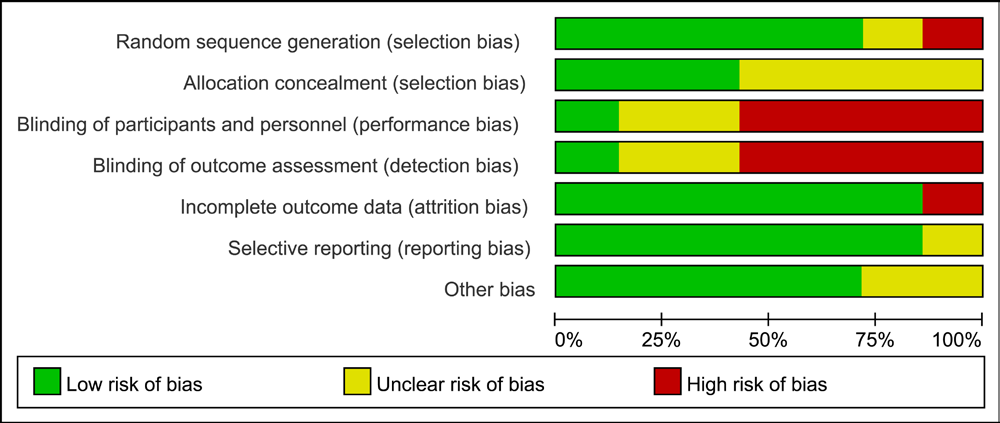
Risk of bias graph: review authors’ judgements about each risk of bias item presented as percentages across all included studies.

Two studies conducted by Zhang (2018) and Deng et al (2018) respectively had a Jadad score of 3,^[23,29]^ 4 studies had a Jadad score of 1 to 2,^[25–28]^ and 1 had a Jadad score of 0.^[24]^ All above are shown in Table 1. On the basis of our definition for good quality, 28% of studies were good quality. The risk of bias of each RCT is shown in Figure 2, and the risk of bias is shown as percentages across all studies in Figure 3. Study conducted by Zhai et al (2010) had a low risk of bias in blinding.^[28]^ Six studies had a high risk of detection.^[23–27,29]^ The risk of performance bias, attribution bias, reporting bias and other bias were high or unclear.

### 3.3. Efficacy of Enterococcus faecium on NEC

**Figure 4.**
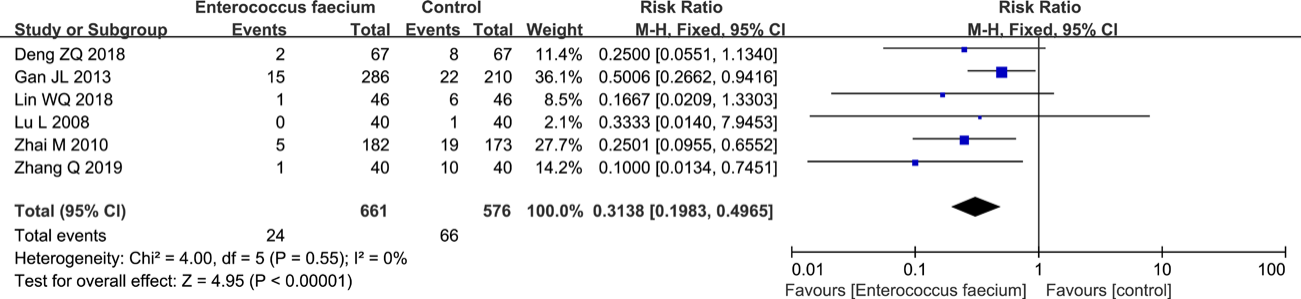
Forest plot of the efficacy of Enterococcus faecium in preventing necrotizing enterocolitis (NEC) in preterm infants.

Six RCTs were included in this meta-analysis to assess the efficacy of Enterococcus faecium in preventing NEC in preterm infants.^[23,25–29]^ The pooled analysis was evaluated by means of a fixed-effects model due to no considerable heterogeneity (*P* = .55, I^2^ = 0%). The outcomes were measured of included studies by means of the incidence of NEC Bell stage II or higher. The pooled incidence was 7.3% (90/1237). The morbidity of NEC Bell stage II or higher indicated significantly difference between Enterococcus faecium groups and control groups (pooled RR: 0.3138, 95% CI: 0.1983–0.4965; Z = 4.95, *P* < .00001; 6 studies, n = 1237, moderate quality evidence, Fig. 4). We downgraded the quality of evidence due to (−1) serious inconsistency. Therefore, Enterococcus faecium could reduce the risk of NEC stage ≥ II of preterm infants.

### 3.4. Incidence of sepsis and infection

**Figure 5.**
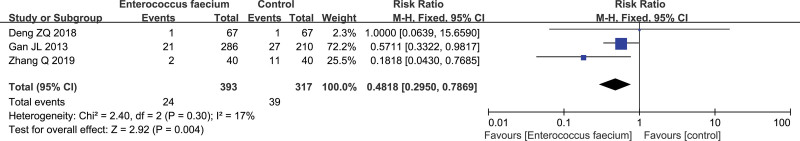
Forest plot of the incidence of infection comparing Enterococcus faecium with placebo in preterm infants.

No data on sepsis was reported. Three studies were used for the analysis of infection in supplement of Enterococcus faecium in preventing NEC in preterm infants.^[23,25,29]^ The pooled outcome was estimated by means of a fixed-effects model by considering no considerable heterogeneity (*P* = .30, I^2^ = 17%). The total morbidity of infection was 8.9% (63/710). As shown in Figure 5, the incidence of infection was significantly lower in the Enterococcus faecium group compared with the control group (RR: 0.4818; 95% CI: 0.2950–0.7869; Z: 2.92, *P*: .004; 3 studies, n = 710, moderate quality evidence, Fig. 5). We downgraded the quality of evidence due to (−1) serious inconsistency. Therefore, Enterococcus faecium could reduce the risk of infection of preterm infants.

### 3.5. Mortality

One of included studies reported the mortality.^[27]^ So, meta-analysis couldn’t be performed. The mortality of control group was 5% (2 of 40 infants), and no death in the Enterococcus faecium group.

### 3.6. Subgroup analysis, sensitivity analysis, and publication bias

**Figure 6.**
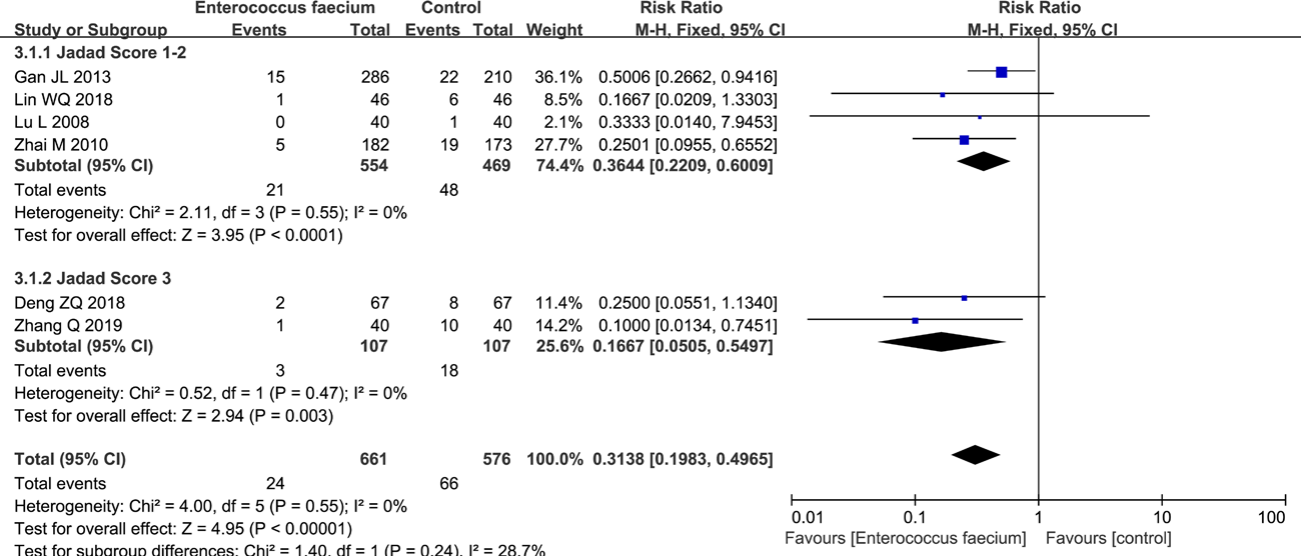
Forest plot of subgroups for the efficacy of Enterococcus faecium in preventing necrotizing enterocolitis (NEC) in preterm infants.

A subgroup analysis was preformed to estimate the efficacy of estimate in preventing NEC in preterm infants. A significant effect of Enterococcus faecium was detected for Jadad score 1 to 2 (RR: 0.3644; 95% CI: 0.2209–0.6009), score 3 (RR: 0.1667; 95% CI: 0.0505–0.5497), Figure 6.

**Figure 7.**
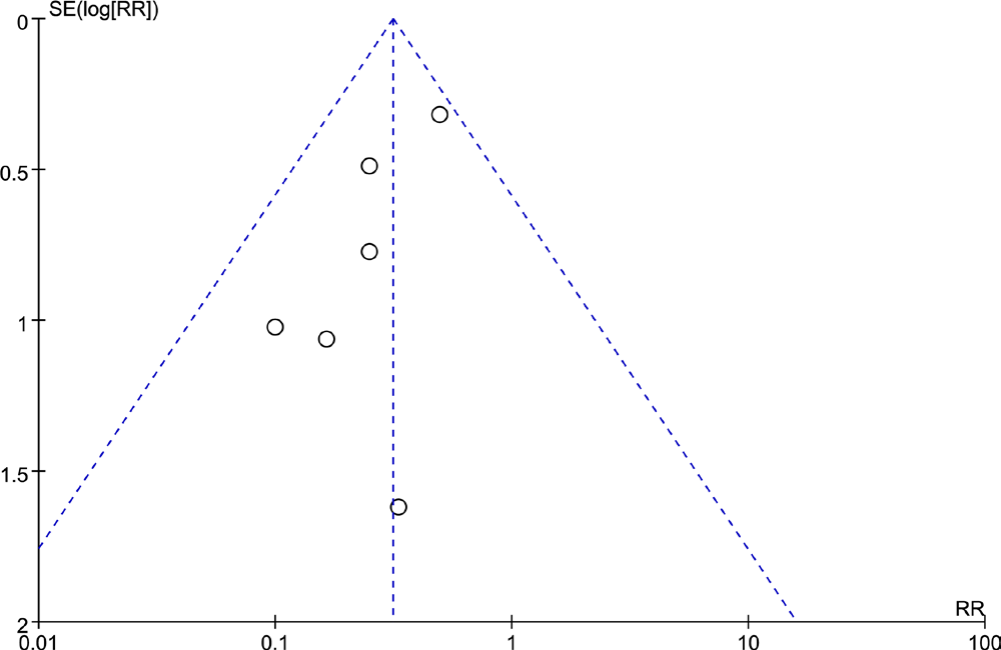
Begg funnel plot for detecting publication bias.

We conducted a sensitivity analysis to estimate the stability of the efficacy of Enterococcus faecium in preventing NEC and to distinguish the source of heterogeneity by removing any study. The efficacy of Enterococcus faecium had high stability and no particular study evidently influenced the pooled result (RR: 0.21, [95% CI: 0.10, 0.41; *P* < .00001]) after removing the study of Gan JL (2013)^[25]^ to 0.35 (95% CI: 0.22, 0.56; *P* < .0001) after removing the study of Zhang Q (2019)^[37]^ (Supplemental Table 1, http://links.lww.com/MD/J504). Furthermore, as the Begg funnel polt shown (Fig. 7), the absence of significant asymmetry indicated the risk of publication bias is small.

## 4. Discussion

NEC is a fatal disease affecting about 7% to 10% of very low BW premature infants (BW < 1500 g), with those BW < 1000 g being at the highest risk.^[4]^ The mortality of infants with NEC was 15% to 40%,^[18,38]^ and the survivors have an increased risk for both short-term and long-term complications including late-onset sepsis, intestinal strictures, short bowel syndrome, cholestasis, and developmental retardation.^[37,39]^ So, it is necessary to prevent NEC in preterm infants, in especial extremely low BW (BW < 1000 g) and extremely premature infants (GA < 28 weeks). Notwithstanding great progresses in preterm infant care have been achieved, up to now, NEC still leads to substantial mortality and medical costs. Annual cost of treating NEC in the U.S.A. amounted to be billions of dollars.^[40]^

The pathogenesis and mechanism of NEC is poorly understood. Although the exact cause of NEC is unknown, it is likely relevant to intestinal inflammation, infection and injury as well as intestinal microbial dysbiosis.^[41]^ The role of probiotics in preventing NEC has been studied and some progress has been made. Preterm infants are at risk of NEC due to intestinal epithelial tissue barrier insufficiency, weak intestinal motility, and immature immune system development.^[14]^ On the basis of some studies supporting the application of probiotics to reduce the incidence of NEC, we hope that more probiotics will be applied to the NICU. Yet, only approximately 10% of NICUs provide probiotics to preterm infants as a strategy to prevent NEC.^[42]^

This systematic review included 6 RCTs and 1 CCT to demonstrate the benefit and safety of Enterococcus faecium for the prevention of NEC in preterm infants. Our results statistically support the conclusion that the Enterococcus faecium could decrease the incidence of NEC compared with the control groups. In addition, we assessed the incidence of infection and death to evaluate safety of Enterococcus faecium. Administration of Enterococcus faecium could cut the risk of infection and death in preterm infants. Therefore, Enterococcus faecium decreases the morbidity of NEC and mortality without increase in incidence of infection. Research performed by Zhang Q (2019)^[29]^ showed that fewer infants in the Enterococcus faecium group (2.5%) developed NEC compared with the control group (25%). Compared with the control group, the incidence of infection in premature infants in Enterococcus faecium group decreased significantly by 27.5% and 5% respectively. It seems that Enterococcus faecium may be safe and effective in prevention of NEC in preterm infants.

This systematic review also had some limitations. Firstly, possibility of industrial bias cannot be excluded. Although there is no restriction on the language of retrieval, all studies included came from China. No literature has been retrieved in other countries. Secondly, the dosages and durations of taking Enterococcus faecium were various. Medilac-Vita (Combined Bacillus Subtilis and Enterococcus faecium Granules with multivitamines, live, made by Beijing Hanmi Pharmaceutical Co., LTD.) was the only probiotics in studies included. The daily dosages ranged from 0.67 to 2.0 g (Medilac-Vita, Enterococcus faecium 1.35 × 10^8^ and Bacillus subtilis 1.5 × 10^7^ per gram). Thirdly, GA and BW of preterm infants were various. The incidence of NEC, infection, and mortality was related to GA and BW. The definition of preterm infants in majority of studies was GA < 37 weeks in this meta-analysis. But Lin WQ et al (2018)^[26]^ limited participants with GA ≤ 34 weeks and BW ≤ 2000 g. Last but not least, the morbidity of NEC and infection, and mortality of preterm infants can be affected by a variety of factors, for instance, the medical level of NICUs.

Based on our results, further research should be carried out and emphasis on the efficacy, timing, dosage and durations of taking probiotics for prevention and treatment of NEC in different GA and BW preterm infants.

## 5. Conclusion

This systematic review and meta-analysis showed that Enterococcus faecium can prevent NEC and reduce the incidence of NEC (Bell stage II or higher) and infection in preterm infants. Enterococcus faecium is effective and safe in preventing NEC in preterm infants.

## Acknowledgments

We express our appreciation to reviewers for their helpful comments on this manuscript.

## Author contributions

**Conceptualization:** Guangguo Men, Lili Wang.

**Data curation:** Guangguo Men, Lili Wang, Xudan Lu, Gang Wen.

**Formal analysis:** Guangguo Men, Gang Wen.

**Funding acquisition:** Guangguo Men.

**Investigation:** Guangguo Men.

**Methodology:** Guangguo Men, Xudan Lu, Qin Lü.

**Project administration:** Guangguo Men, Lili Wang.

**Resources:** Guangguo Men.

**Software:** Xudan Lu.

**Writing – original draft:** Guangguo Men.

**Writing – review & editing:** Guangguo Men, Gang Wen, Qin Lü.

## Supplementary Material


