# Supplementary material for: Can Enterococcus faecium prevent NEC in preterm infants?: A systematic review and meta-analysis
Source: Medicine (Baltimore). 2023 Aug 11;102(32):e34787. doi: 10.1097/MD.0000000000034787 (PMC10419755; doi:10.1097/MD.0000000000034787)
Supplement: Supplementary file 1 [file medi-102-e34787-s001.pdf]

Supplemental Table 1. Sensitivity analysis results of the efficacy of Enterococcus faecium in preventing NEC in preterm infants.

Omitted studies	Pooled results				Heterogeneity		
	RR	95% CI	*P* value		I^2	*p* value	Analytical effect model
Deng ZQ 2018[36]	0.32	0.20,0.52	<0.00001		0%	0.43	Fixed-effect model
Gan JL 2013[35]	0.21	0.10,0.41	<0.00001		0%	0.84	Fixed-effect model
Lin WQ 2018[33]	0.33	0.20,0.52	<0.00001		0%	0.48	Fixed-effect model
Lu L 2008[34]	0.31	0.20,0.50	<0.00001		0%	0.41	Fixed-effect model
Zhai M 2010[31]	0.34	0.20,0.57	<0.0001		0%	0.48	Fixed-effect model
Zhang Q 2019[32]	0.35	0.22,0.56	<0.0001		0%	0.67	Fixed-effect model
